# Acrylamide Reduction Strategy in Combination with Deoxynivalenol Mitigation in Industrial Biscuits Production

**DOI:** 10.3390/toxins11090499

**Published:** 2019-08-27

**Authors:** Michele Suman, Silvia Generotti, Martina Cirlini, Chiara Dall’Asta

**Affiliations:** 1 Advanced Research Laboratory, Barilla G. e R. Fratelli S.p.A., Via Mantova, 166-43122 Parma, Italy; 2 Department of Food & Drug, University of Parma, Parco Area delle Scienze, 95/A-43124 Parma, Italy

**Keywords:** acrylamide, deoxynivalenol, multiple mitigation strategies, design of experiments, bakery food processing, biscuits

## Abstract

Acrylamide is formed during baking in some frequently consumed food products. It is proven to be carcinogenic in rodents and a probable human carcinogen. Thus, the food industry is working to find solutions to minimize its formation during processing. To better understand the sources of its formation, the present study is aimed at investigating how acrylamide concentration may be influenced by bakery-making parameters within a parallel strategy of mycotoxin mitigation (focusing specifically on deoxynivalenol—DON) related to wholegrain and cocoa biscuit production. Among Fusarium toxins, DON is considered the most important contaminant in wheat and related bakery products, such as biscuits, due to its widespread occurrence. Exploiting the power of a Design of Experiments (DoE), several conditions were varied as mycotoxin contamination levels of the raw materials, recipe formulation, pH value of dough, and baking time/temperature; each selected treatment was varied within a defined range according to the technological requirements to obtain an appreciable product for consumers. Experiments were performed in a pilot-plant scale in order to simulate an industrial production and samples were extracted and analysed by HPLC-MS/MS system. Applying a baking temperature of 200 °C at the highest sugar dose, acrylamide increased its concentration, and in particular, levels ranged from 306 ± 16 µg/Kg d.m. and 400 ± 27 µg/Kg d.m. in biscuits made without and with the addition of cocoa, respectively. Conversely, using a baking temperature of 180 °C in the same conditions (pH, baking time, and sugar concentrations), acrylamide values remained below 125 ± 14 µg/Kg d.m. and 156 ± 15 µg/Kg d.m. in the two final products. The developed predictive model suggested how some parameters can concretely contribute to limit acrylamide formation in the final product, highlighting a significant role of pH value (correlated also to sodium bicarbonate raising agent), followed by baking time/temperature parameters. In particular, the increasing range of baking conditions influenced in a limited way the final acrylamide content within the parallel effective range of DON reduction. The study represents a concrete example of how the control and optimization of selected operative parameters may lead to multiple mitigation of specific natural/process contaminants in the final food products, though still remaining in the sensorial satisfactory range.

## 1. Introduction

Process-related mitigation strategies are among the most promising tools for controlling and minimizing mycotoxins, i.e., Fusarium mycotoxins, in cereal-based products [1]. Among Fusarium mycotoxins, deoxynivalenol (DON), along with its modified forms, is considered the most important contaminant in wheat and related bakery products, such as biscuits, due to its widespread occurrence [2]. It has been shown that milling and baking/roasting may effectively reduce the amount of DON in the bakery. At this purpose, Generotti et al. reported on the strategic mitigation of DON and its related compounds deoxynivalenol-3-glucoside (DON3Glc) and culmorin, during two different biscuit processes, exploiting the synergistic effects of recipe formulation and thermal treatment [3]. A significant reduction was achieved while not negatively impacting product quality and identity. However, increased time and temperature during baking might favor the formation of process-derived contaminants, such as acrylamide, in the final product.

Besides often reported among the most studied contaminants in cereal-based products, mycotoxins and acrylamide are almost unavoidable compounds, being the former natural toxins accumulated in crops under natural field conditions, and the latter process-related compounds formed in food during manufacturing, as a consequence of chemical reaction triggered by processing.

Although their toxic effects have been deeply studied as single compounds, little to nothing is known about combined effects exerted in animals and humans. Nonetheless, the scientific community is posing increasing emphasis on health concern related to the exposure to chemical mixtures, as recently reported by the European Food Safety Authority [4]. Therefore, the identification of possible strategies for the simultaneous mitigation of mycotoxins and acrylamide, are of upmost interest for the agro-food sector.

Concerning process-related compounds, food industry and the European Commission have undertaken extensive efforts since 2002, when scientists from the Swedish National Food Authority and the University of Stockholm reported high levels of acrylamide in normally cooked starch-rich food (compared to what had been reported earlier in other food commodities), in order to investigate pathways of formation and to reduce its levels in processed food.

Acrylamide is typically formed from an amino acid, primarily asparagine, and a reducing sugar such as fructose or glucose in starchy food products during high temperature cooking, including frying, baking and roasting through a series of reactions, known as Maillard reactions. Its formation starts at temperatures around 120 °C and peaks at temperatures between 160 and 180 °C [5,6]. Due to its toxicity and possible carcinogenic effects to humans (IARC—International Agency for Research on Cancer [7]), the European Commission has established mitigation measures and benchmark levels for its concrete reduction in food [8,9].

Several attempts have been made so far to develop mitigation strategies for acrylamide formation in bakery products, mainly focused on the processing stage and recipes [10,11,12,13,14,15,16,17]. Each strategy could present limiting factors in their applicability depending on the product type and industrial settings as feasibility and compatibility with processing, formulation, impact on sensory and nutritional characteristics, regulatory compliance and costs.

Acrylamide formation is favored by a high baking temperature and time treatment [17,18,19,20,21,22,23], therefore a decreasing of thermal input represents an effective way of mitigation. Reduction can be obtained by applying prolonged heating at lower temperatures, or at lower pressure than the atmospheric one [16] or by optimizing the oven temperature profile. On the other hand, a decreased thermal input may significantly affect the achievement of both appropriate hygienic properties and sensorial acceptance of the final product.

**Figure 1:**
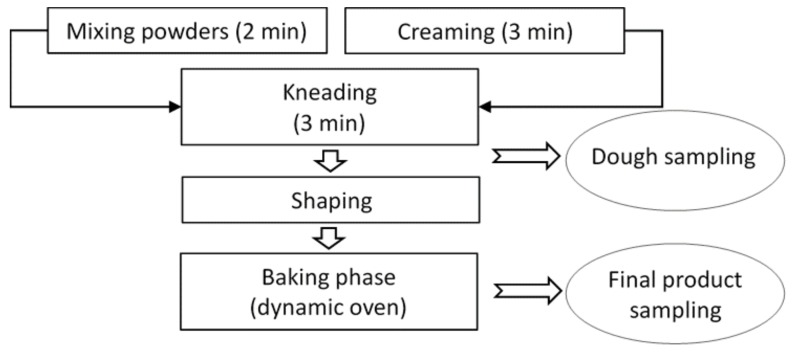
Scheme of wholegrain and cocoa biscuit production.

Moving from our previous studies [24,25,26,27], the present investigation is aimed at verifying how acrylamide concentration in bakery products, such as wholegrain and cocoa biscuits, is affected by modifications of technological parameters (recipe formulation and baking time/temperature) during biscuit-making process (Figure 1), while at the same targeting potential DON reduction and without affecting the sensory properties.

In this regard, predictive models would represent a time and cost-saving tool for finding the most suitable conditions for minimizing both natural occurring and process-related contaminants. In the present work, we have exploited them to estimate acrylamide evolution in a considered system and to manage the industrial process to optimize the role of involved parameters with regard to its formation.

Starting from naturally DON contaminated raw material, the experiments were performed using statistical Design of Experiment (DoE) schemes to explore the relationship between the analytical responses and independent variables conducting to an overall optimization of the baking process [28]. In particular, this approach was conducted in order to consider only those modifications that can be really applied to the industrial scale, obtaining a final product appreciable by consumers and remaining in an acceptable technological range.

## 2. Results and Discussion

The present study was carried out to better understand whether and how acrylamide evolution could be influenced by selected technological factors within a strategy of DON mitigation related to wholegrain and cocoa biscuit production. Different parameters were considered and a variation range was defined, as shown in Table 1. The statistical model required 19 single experiments per technological process; acrylamide level was measured by LC-MS/MS according to a previously published method with slight modifications [29].

### 2.1. Statistical Elaboration of the Experimental Model

**Figure 2:**
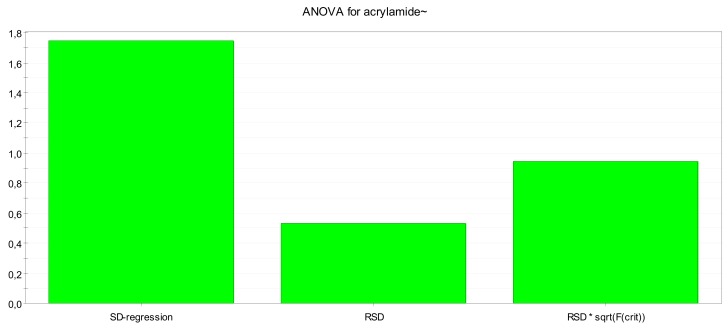
Design of Experiments on acrylamide levels within cocoa biscuit-making—Model Robustness by ANOVA plot.

At the end of the analysis performed on the collected samples, MODDE software extracted an answer concerning robustness and prediction capability of the method, evaluating model efficiency by fitting (R2) and prediction (Q2) values. Replicate values of acrylamide reduction per experiment were expressed on dry matter basis and averaged for the statistical elaboration. Partial least-squares (PLS) was chosen as the statistical regression treatment. The two statistical models (wholegrain and cocoa biscuit model) gave high fitting (*R*2 > 0.7) and good prediction value (Q2 ≥ 0.5), referring to the MODDE software output settings. Model robustness was also confirmed by ANOVA plot (Figure 2), being standard deviation of the regression much larger than standard deviation of the residuals.

### 2.2. Acrylamide Evolution within Biscuit-Making Technology

**Figure 3:**
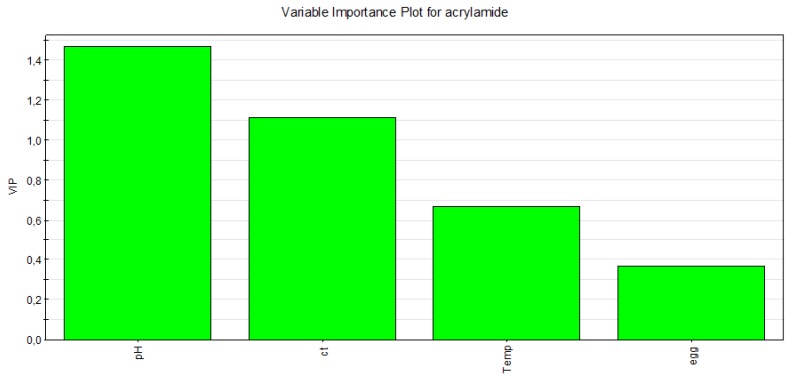
Variable Importance Plot (VIP) obtained for the data referred to cocoa biscuit-making process: influence of each main factor with respect to acrylamide response: pH, ct (cooking time), Temperature (Temp), Egg.

All values were collected in a Variable Importance Plot (VIP, Figure 3) that enables (and illustrates in an effective and condensed way) an understanding of the effect of each factor in terms of influence on acrylamide response. 

In the present case, VIP would suggest that the pH value, has the most relevant effect on the final acrylamide level: in fact pH increase is responsible for an acceleration of the reaction between asparagine and the reducing sugars, followed by baking time/temperature parameters. 

With regard to the other minor ingredients, dextrose (or glucose) content confirm (as reported in previous scientific literature findings) to contribute to the overall acrylamide increase. When high dextrose level and higher thermal input are employed (200 °C for 8 min) an acrylamide increase up to 120% was observed (data not shown) with respect to the central point. On the other hand, a combination of lower dextrose content and moderate thermal input (180 °C for 8 min) may lead to a reduction up to 77%.

This is consistent with literature studies in which it was demonstrated that increasing the quantity of sugars in cookies formulation, as glucose and sucrose, the concentration of acrylamide raised up especially when a temperature of 205° was applied for 11 min. Moreover, the choice of the sugar resulted crucial for keeping acrylamide formation under control. Using glucose, a reducing compound, and applying the same baking conditions, acrylamide content increased more in respect to sucrose. So, the authors suggested to use sucrose in order to obtain a reduction of about the 50% of acrylamide production [30,31]. Similar results were showed by Vass et al. [32] whom replaced invert sugar syrup with sucrose in wheat crackers, obtaining an acrylamide reduction by 60%. In addition, the same studies speculated that applying baking temperatures of 160°C the formation of acrylamide remains below 150 µg/Kg [30]. Also in this study, the measured amount of acrylamide presented values below or close to 150 µg/Kg when the baking temperature did not exceed 180 °C (Table 2). 

Furthermore, some authors showed how acrylamide mitigation can be achieved by adding amino acids or protein-based ingredients to food, which may influence the reaction pathway or favor AA degradation [33,34,35]. In our study, no significant mitigation was obtained probably due to the close variation range related to milk/egg content in the recipe formulation.

Basically, acrylamide content seems to be affected by the food matrix, being higher for the cocoa biscuits than for the wholegrain biscuits. This could be related to the additional acrylamide load related to cocoa beans roasting (Table 2). It was indeed demonstrated that acrylamide could be present in roasted cocoa beans in the order of mg/Kg and the content may depend form the temperature used during roasting [36].

With regard to the sodium bicarbonate content and the correspondent pH variation, a potential reduction in acrylamide level higher than 50% could be achieved in the finished product, remaining within an acceptable range from the sensorial point of view. Sodium bicarbonate could indeed limit the formation of acrylamide if compared to other raising agents as ammonium hydrogen carbonate. This was demonstrated during experiments conducted on biscuits, in which also the addition of tartaric and citric acids was tested in order to reduce acrylamide content. The authors showed that a reduction of about 70% of acrylamide content was achieved when sodium bicarbonate was applied [31]. 

Taking into account the previously mentioned mycotoxin mitigation strategy, baking time/temperature play an important role in order to achieve a parallel significant DON mitigation; considering experiments carried out within the most severe time/temperature baking conditions, the greatest effect was observed with the baking step being performed at 200 °C for 8 min. In particular, an increase in time during the baking phase, in an acceptable technological range, can effectively reduce DON content in the final product [3].

Notably, an acrylamide reduction ranged from 77% to 100% was achieved in the finished product when baking was conducted at 180 °C for 5 min, though still remaining in the sensorial satisfactory range.

**Figure 4:**
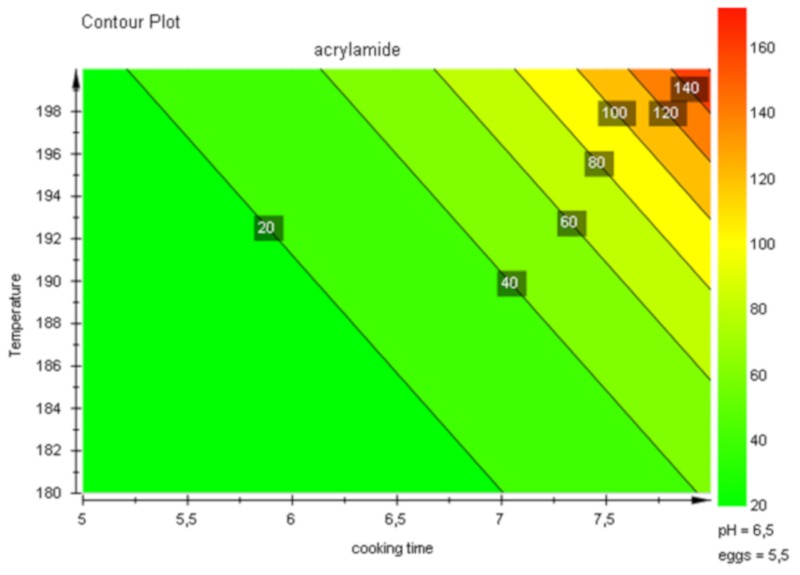
Contour Plot: delta-acrylamide (predicted increase) values of the cocoa biscuit-making experiments; Temperature vs. cooking time.

Overall, data collected within this study allow to proper set the baking time and temperature for controlling mycotoxin and acrylamide content in the finished product, as suggested by the response Contour Plot (Figure 4). The increase of baking parameters (which also goes in the direction of the obvious industrial requirement to speed-up the process, in order to increase the productivity as much as possible) within a range of mycotoxin mitigation (up to 20% of reduction) affects in a restricted manner the final acrylamide content (difference of the values of acrylamide expressed as predicted increase, named “delta acrylamide” in the figure caption), without implications on the organoleptic properties and consumer safety. 

As a major outcome of this study, this allows one to design proper synergistic mitigation strategies for multiple contaminants along the food production chain. 

## 3. Materials and Methods

### 3.1. Chemicals

Methanol and formic acid (p.a.), both HPLC gradient grade, were obtained from BDH VWR International Ltd. (Poole, UK). Acetonitrile was purchased from J.T. Baker (Deventer, The Netherlands) and ammonium acetate (MS grade) and glacial acetic acid (p.a.) were obtained from Sigma-Aldrich (Vienna, Austria). Standard acrylamide solution was purchased from Sigma-Aldrich (Milan, Italy). Acrylamide internal standard (13C3-acrylamide, 1 mg/mL in methanol) was obtained from Cambridge Isotope Laboratories, Inc. (Andover, MA, USA). Deionized water was used for all procedures. Water was purified successively by reverse osmosis and a Milli-Q plus system from Millipore (Molsheim, France). Deoxynivalenol standard was obtained from RomerLabs^®^Inc. (Tulln, Austria). OASIS^®^ HLB 3 cc (60 mg) extraction cartridges were purchased from Waters (Manchester, UK). Glass vials with septum screw caps were purchased from Phenomenex (Torrance, CA, USA). Centrifugal filter units (Ultrafree MC 0.22 mm, diameter 10 mm) were obtained from Millipore (Billerica, MA, USA).

### 3.2. Biscuit-Making Production in Pilot-Plant Scale

Pilot-plant scale experiments were performed according to a previously published study [3]. Briefly, three batches of wheat bran naturally infected with Fusarium spp. were analysed with a focus on DON, selected and mixed with a blank wheat flour for the wholegrain biscuit production. Concerning cocoa biscuit trials, the same three mix flours were employed and three different batches of cocoa powder were selected. 

Different doughs were prepared in order to obtain a final dough of about 1000 ± 30 g; regarding wholegrain biscuits, the ingredients were wheat flour (60%), bran (7%), cream of tartar, glucose syrup, and salt. Eggs, margarine, and dextrose were added depending on the value reported in recipes obtained from the experimental design (Table 3). Sodium bicarbonate was added in order to reach the appropriate pH value. Water amount ranged from 1.6% to 5%, depending on the technological requirements. 

In order to produce cocoa biscuits, 45% of wheat flour, 7% of bran, 4% of cocoa powder, margarine, glucose syrup, and salt were employed. Milk and dextrose amount were indicated in the model generated by Design of Experiment (Table 4). Sodium bicarbonate was added in order to reach the appropriate pH value. The optimal amount of water to be added to each dough sample was established on the basis of internal technological knowledge.

The process for wholegrain and cocoa biscuit production consisted essentially of the following steps: creaming, dough preparation, and baking step. Firstly, wheat flour was mixed with all solid powder ingredients using a test planetary kneader for 2 min. Dextrose and margarine were mixed separately by using another test planetary kneader for 3 min (creaming step). At a later stage, cream and powders were mixed together for 3 min. Dough was shaped and rounded pieces of about 4 cm diameter (approximately 10 g) were obtained from dough and rested for 10 min at room temperature. Baking step was performed in a pilot-scale dynamic oven (Tagliavini, Parma, Italy). The overall process is summarized in Figure 1. Nineteen different tests for each process were performed (Table 3 and Table 4, respectively). 

Acrylamide content was examined in mix powders, before and after baking process. Before the acrylamide content analysis, samples were stored at −20 °C. Each sample was extracted and analyzed in duplicate.

### 3.3. Moisture Content Determination

The moisture contents of mix flours, doughs, and baked products were measured by taking a 5 g ground sample and heating it in a thermostatic oven at 105 °C for 6 h. All the results were compared on a dry matter (d.m.) basis.

### 3.4. Experimental Design and Statistical Evaluation

Design of Experiments (DoE) is used in many industrial issues, in the development and optimization of manufacturing processes, making a set of experiments representative with regards to a given question. DoE is a series of tests in which purposeful changes are made to the input variables of a system or process and the effects on response variables are measured: the analyst is interested in studying the synergistic effects of some interventions (the “treatments”) to optimize the final process [28].

Among the wholegrain and cocoa biscuit-making parameters, several conditions were varied during the experiments: DON contamination level on wheat bran; dextrose, margarine, egg and milk content (as percentage in recipes); pH value (as sodium bicarbonate content) and baking time and temperature. Each selected treatment was varied within a range defined according to the technological requirements to obtain an organoleptically appreciable product for consumers: the central experimental values, indicated in Table 1, represent the optimal combination of ingredients/recipe and operative conditions that permit to achieve the most appropriate finished product.

Experimental data were then analysed by a multi-variate analysis approach based on the partial least-squares (PLS) technique, using a dedicated statistical package (MODDE software, version 9.1, 2012; Umetrics, Umea, Sweden).

### 3.5. Sample Extraction and Instrumental Conditions—Deoxynivalenol

Concerning Deoxynivalenol, samples were extracted according to a previously published procedure [3,25] with slight modifications. Briefly, a total of 10.00 g of flour or dough or biscuit sample were extracted with 100 mL of an acetonitrile/water (84:16, *v*/*v*) mixture by homogenization at a medium-to-high speed for 2 min using a mixer (Oster, New York, USA). The extract was allowed to settle for 15 min. Afterwards, 5 mL were poured into a 10 mL vial, and evaporated to dryness under a nitrogen stream. The extract was reconstituted with 100 mL of 13C-DON internal standard solution (100 ng/mL in methanol) and 900 mL of water. Each extraction cartridge column was activated using 2 mL of methanol, and 2 mL of methanol:water (10:90, *v*/*v*). The sample extract was then slowly passed through the OASIS^®^ HLB 3 cc (60 mg) (Waters, Manchester, UK) column using a vacuum chamber system. A solution of methanol:water (20:80, *v*/*v*) was used for washing, followed by elution with 1 mL of methanol. The eluate was evaporated under a gentle stream of nitrogen, and the residue was dissolved in 200 mL of eluent A (methanol:water, 20:80 *v*/*v*, 0.5% acetic acid, and 1 mM ammonium acetate) prior to UHPLCMS/MS analysis. Ultrahigh-performance liquid chromatography (UHPLC) was performed using a Dionex Ultimate^®^ 3000 LC systems (Thermo Fisher Scientific Inc., Waltham, MA, USA) and a Kinetex Biphenyl column (2.6 mm; 100 × 2.10 mm; Phenomenex). The flow rate of the mobile phase was 400 mL/min, and the injection volume was 20 mL. The column oven was set to 30 °C. A linear binary gradient composed of (A) water (0.5% acetic acid, 1 mM ammonium acetate) and (B) methanol (0.5% acetic acid, 1 mM ammonium acetate) was employed. The gradient was as follows: 0–4 min to 40% B; 4–20 min to 80% B; 20–22 min, isocratic step 80% B; finally, a re-equilibration step at 10% B (the initial value) was performed for another 3 min, bringing the total analysis time to 25 min. Before UHPLC-MS/MS analysis, all samples were filtered through centrifugal filter units for clarification. ESI-MS/MS was carried out by a Q-Exactive (Thermo Fisher Scientific Inc., Waltham, MA, USA) mass spectrometer. Experiments were performed in full MS data scan for quantification and data-dependent scan with the following settings: the capillary temperature was set to 300 °C; the sheath gas and auxiliary gas flow rates were set to 40 and 10 units, respectively; the spray voltage was set to 3500 kV; the S-lens RF level was set to 55 V. All equipment control and data processing were performed by Excalibur software (Thermo Fisher Scientific Inc., Waltham, MA, USA). Deoxynivalenol measurements in all the samples were performed using isotopically labeled standard and calibration vs. matrix-matched standards.

### 3.6. Sample Extraction and Instrumental Conditions—Acrylamide

Sample extraction for acrylamide was performed according to a previously published procedure [29] with slight modifications. Briefly, samples were finely ground in a blender to homogeneity before extraction. 1 g of sample was weighed into a polypropylene graduated conical tube and different volumes of a 300 µl ml-1 internal standard solution (13C3-labeled acrylamide in 0.1% (*v*/*v*) formic acid) followed by 10 mL 0.1% (*v*/*v*) formic acid were added on the base of the acrylamide concentrations supposed to be present in the samples. After mixing for 10 min on a vortexer the extract was centrifuged at 1,0000 rpm for 5 min. A 3-mL portion of clarified solution was removed avoiding to collect top oil layer when present and filtered through a 0.45 µm nylon syringe filter (Phenomenex, Torrance, CA, USA) before injection into the HPLC-MS/MS system (injection volume 10 µL). LC-ESI-MS/MS in positive ion mode analysis was achieved using a Surveyor LC quaternary pump separation system (Thermo Fisher Scientific Inc.) coupled to a linear ion trap LXQ mass spectrometer (Thermo Fisher Scientific Inc., Waltham, MA, USA). 

Chromatographic separation was performed using a Synergi Hydro-RP (150 × 2.0 mm) 4 µm analytical column (Phenomenex, Torrance, CA, USA). Elution was carried out at a flow rate of 0.2 mL/min, in isocratic conditions, at 30 °C using as mobile phase a mixture of 98.9% water, 1% methanol and a.1% formic acid (*v*/*v*/*v*). In these conditions, the retention time of acrylamide was about 4 min. A time programmed valve was used to discard the eluate from the column for the first 2.5 min in order to eliminate the compounds with retention times shorter than acrylamide. At 8 min the column flow was again diverted and the mobile phase changed to 100% methanol in order to clean the column from strongly retained compounds within a total run time of 10 min. MS/MS conditions were set as follows: capillary temperature was set to 160 °C; the sheath gas was set to 35 units; the spray voltage was kept at 4500 V; capillary voltage was kept at 9 V. All parts of the equipment and data processing were performed by the computer software Xcalibur (Thermo Fisher Scientific Inc.). MS/MS analysis was carried out by selecting the ions at *m*/*z* 72 and *m*/*z* 75 as precursor ions for acrylamide and 13C3-acrylamide respectively. 

The area of the chromatographic peaks of the extracted ion at *m*/*z* 55, due to the transition 72 > 55, and at *m*/*z* 58, due to the transition 75 > 58 were used for the quantitative analysis. The quantitative analysis was carried out with the method of the internal standard. The relative response factor of acrylamide with respect to 13C3-acrylamide was calculated daily by analysing a standard solution.

## 4. Conclusions and Outlook

Since precursors of acrylamide are present in the dough, modifications in recipe formulation and time-temperature control during baking process could be actually used to reduce acrylamide content in biscuits. 

In the present study, the influence exerted by modifying ingredients and industrial conditions on acrylamide levels within a parallel mycotoxin mitigation strategy was investigated. 

The obtained processing models showed a good fitting, robustness, and prediction capability, suggesting the most significant parameters. These can concretely contribute to the reduction of acrylamide levels in the final food product. 

Acrylamide formation is evidently baking time- and temperature-dependent, therefore prolongation of heat treatments results in higher contents of acrylamide; however, when such parameters are moved within the optimal range for DON mitigation, the actual increase affects, in a limited way, the final acrylamide content without significant implications on the organoleptic properties.

In conclusion, the present report demonstrates the effectiveness of a careful design of process parameters for the mitigation of multiple contaminants in the final product, thus remaining within the consumer’s sensorial acceptance.

## Figures and Tables

**Table 1:** Wholegrain and cocoa biscuit experiments—pilot plant processing conditions.

TREATMENT	WHOLEGRAIN BISCUIT MAKING			COCOA BISCUIT MAKING		
	Minimum	Central Point	Maximum	Minimum	Central Point	Maximum
DON bran level (µg/kg)	600	1050	1500	600	1050	1500
Dextrose (%)	15	19	23	15	19	23
Milk (%)	-	-	-	5	6.5	8
Eggs (%)	4	6	7	-	-	-
Margarine (%)	10	15	20	-	-	-
pH value	5	6.5	8	5	6.5	8
Baking time (min)	5	6.5	8	5	6.5	8
Baking temperature (°C)	180	190	200	180	190	200

**Table 2:** Analytical results for acrylamide levels throughout several wholegrain and cocoa biscuit-making process trials.

Sample	Baking Stage					
	DON in Wholegrain Flour (µg/kg d.m.) *	NaHCO3 (g)	Time (min)	Temperature (°C)	Acrylamide (µg/kg d.m.) *	DON (µg/kg d.m.) *^§^
Wholegrain biscuits	219 ± 8	8	5	180	16 ± 3	154 ± 1
	304 ± 9	8	8	180	125 ± 14	192 ± 4
	219 ± 8	8	5	200	66 ± 10	188 ± 7
	304 ± 9	8	8	200	306 ± 16	189 ± 7
Cocoa biscuits	219 ± 8	8	5	180	43 ± 13	129 ± 7
	304 ± 9	8	8	180	156 ± 15	208 ± 2
	219 ± 8	8	5	200	185 ± 15	154 ± 1
	304 ± 9	8	8	200	400 ± 27	192 ± 4

* Data expressed as mean value ± standard deviation. ^§^ Data reported and discussed elsewhere [3].

**Table 3:** Experimental data set for screening variables effects on acrylamide levels within the wholegrain biscuit-making process steps: full-factorial central composite design.

Experiment Number	DON in Bran (µg/kgd.m.) ^1^	Dextrose (%)	Margarine (%)	pH	Eggs (%)	Baking Stage	
						Time (min)	Temperature (°C)
1	600 ± 16	15	10	5	4	5	180
2	1500 ± 92	15	10	5	7	5	200
3	600 ± 16	23	10	5	7	8	180
4	1500 ± 92	23	10	5	4	8	200
5	600 ± 16	15	20	5	7	8	200
6	1500 ± 92	15	20	5	4	8	180
7	600 ± 16	23	20	5	4	5	200
8	1500 ± 92	23	20	5	7	5	180
9	600 ± 16	15	10	8	4	8	200
10	1500 ± 92	15	10	8	7	8	180
11	600 ± 16	23	10	8	7	5	200
12	1500 ± 92	23	10	8	4	5	180
13	600 ± 16	15	20	8	7	5	180
14	1500 ± 92	15	20	8	4	5	200
15	600 ± 16	23	20	8	4	8	180
16	1500 ± 92	23	20	8	7	8	200
17	1050 ± 48	19	15	6.5	6	6.5	190
18	1050 ± 48	19	15	6.5	6	6.5	190
19	1050 ± 48	19	15	6.5	6	6.5	190

^1^ Data expressed as mean value ± standard deviation.

**Table 4:** Experimental data set for screening variables effects on acrylamide levels within the cocoa biscuit-making process steps: full-factorial central composite design.

Experiment Number	DON in Bran (µg/kgd.m.) ^1^	Dextrose (%)	Milk (%)	pH	Baking Stage	
					Time (min)	Temperature (°C)
1	600 ± 16	23	5	5	5	180
2	600 ± 16	15	8	8	5	180
3	1500 ± 92	15	5	5	8	180
4	1500 ± 92	23	8	8	8	180
5	600 ± 16	23	5	8	5	200
6	1500 ± 92	23	8	5	5	200
7	1500 ± 92	15	5	8	8	200
8	600 ± 16	15	8	5	8	200
9	1500 ± 92	23	5	8	5	180
10	600 ± 16	23	8	5	5	180
11	600 ± 16	15	5	8	8	180
12	1500 ± 92	15	8	5	8	180
13	600 ± 16	15	5	5	5	200
14	1500 ± 92	15	8	8	5	200
15	1500 ± 92	23	5	5	8	200
16	600 ± 16	23	8	8	8	200
17	1050 ± 48	19	6.5	6.5	6.5	190
18	1050 ± 48	19	6.5	6.5	6.5	190
19	1050 ± 48	19	6.5	6.5	6.5	190

^1^ Data expressed as mean value ± standard deviation.
